# Pharmacokinetics, safety, and efficacy of daridorexant in Japanese subjects: Results from phase 1 and 2 studies

**DOI:** 10.1111/jsr.14302

**Published:** 2024-08-08

**Authors:** Makoto Uchiyama, Kazuo Mishima, Tomoko Yagi, Tatsuya Yoshihara, Takashi Eto, Clemens Muehlan, Osamu Togo, Yuichi Inoue

**Affiliations:** ^1^ Department of Psychiatry Nihon University School of Medicine Tokyo Japan; ^2^ Tokyo Adachi Hospital Tokyo Japan; ^3^ Department of Neuropsychiatry Akita University Graduate School of Medicine Akita Japan; ^4^ Kurume University School of Medicine Fukuoka Japan; ^5^ SOUSEIKAI Fukuoka Mirai Hospital Clinical Research Center Fukuoka Japan; ^6^ SOUSEIKAI Hakata Clinic Fukuoka Japan; ^7^ Clinical Pharmacology Idorsia Pharmaceuticals Ltd Allschwil Switzerland; ^8^ Data Management & Biometry, Nxera Pharma Japan Co., Ltd Tokyo Japan; ^9^ Yoyogi Sleep Disorder Center Tokyo Japan

**Keywords:** daridorexant, elderly, insomnia, Japanese, orexin receptor antagonist, pharmacokinetics

## Abstract

Daridorexant is a dual orexin receptor antagonist for the treatment of insomnia. We report results from the first two randomised, double‐blind clinical studies of daridorexant in Japanese subjects. In the Phase 1 study, daridorexant (10, 25, 50 mg) or placebo were administered in the morning for 4 days in 24 young (mean age 26.9 years) and 24 older (mean age 69.7 years) healthy Japanese adults. Daridorexant reached a peak plasma concentration within 1.0 h across every dose and age group. For all doses, the mean plasma concentration of daridorexant showed a similar change between the age groups. Exposure parameters increased dose‐dependently with minimal/no accumulation upon repeated dosing. The terminal half‐life was ~8 h. In the Phase 2, four‐period, four‐way crossover study, 47 Japanese subjects (mean age 50.4 years) with insomnia disorder were randomised to receive four treatments (daridorexant 10, 25, 50 mg, placebo) during four treatment periods, each consisting of two treatment nights (5–12 day washout between treatment periods). Subjects continued their fourth treatment for 12 further days. A statistically significant dose–response relationship (multiple‐comparison procedure‐modelling, *p* < 0.0001) was found in the reduction of polysomnography‐measured wake after sleep onset (WASO; primary endpoint) and latency to persistent sleep (secondary endpoint) from baseline to days 1/2. Statistically significant dose–response relationships were also observed for secondary subjective endpoints from baseline to days 1/2 (sWASO, latency to sleep onset). All daridorexant doses were well tolerated, with no treatment discontinuations and no next‐morning residual effects. These results supported further investigation of daridorexant in Japanese patients with insomnia disorder.

## 1 INTRODUCTION

Insomnia disorder, characterised by the long‐standing difficulty in initiating and/or maintaining sleep with a substantial impairment of daytime functioning (American Psychiatric Association, 2013), is a common disorder in Eastern Asian and Western populations (Cao et al., 2017; Kim et al., 2000; Roth, 2007). In patients with insomnia, functional neuroimaging studies show that wake‐promoting areas in the brain may be overly active during the night, leading to the hypothesis that the inability to fall or stay asleep may be related to a failure of arousal mechanisms to decline in activity from waking to sleep states (Buysse et al., 2011; Nofzinger et al., 2004). Recently, some experts have suggested that in patients with insomnia, a hyperactive orexin system may contribute to the functional imbalance in the arousal and sleep circuits (Palagini et al., 2023). In 2007, the sleep promoting effects of the dual orexin receptor antagonist (DORA) almorexant was shown for the first time across several species, including human (Brisbare‐Roch et al., 2007). This was followed by a wealth of data indicating that by suppressing the wakefulness during the nocturnal sleep period without causing inhibition of the central nervous system, DORAs could promote sleep without the associated side effects that are intrinsic to non‐benzodiazepine gamma‐aminobutyric acid type A (GABA_A_) receptor modulators (Z‐drugs) (Griffin 3rd et al., 2013; Ramirez et al., 2013; Tannenbaum et al., 2014; Tannenbaum et al., 2016). DORAs have since emerged as a new pharmaceutical class for the treatment of insomnia disorder (Herring et al., 2016; Mignot et al., 2022; Rosenberg et al., 2019).

Daridorexant is a DORA approved for the treatment of insomnia in adults in the USA, EU, the UK, Canada, and Switzerland. Daridorexant has shown a rapid absorption enabling a fast sleep onset, an optimised elimination profile enabling sleep maintenance during the night without next‐morning hangover and no drug accumulation with repeated nightly dosing (Muehlan et al., 2018; Muehlan et al., 2019). Consistent with the pharmacokinetic (PK) profile of daridorexant, in Phase 2 and 3 studies conducted primarily in Western populations, daridorexant (50 and 25 mg) significantly improved objective sleep onset, sleep maintenance, and self‐reported total sleep time (sTST), with the highest dose (50 mg) being the most efficacious (Dauvilliers et al., 2020; Mignot et al., 2022; Zammit et al., 2020). In one of the Phase 3 studies, daridorexant 50 mg also improved daytime functioning with no evidence of next‐morning hangover (Mignot et al., 2022). Subgroup analyses indicated similar efficacy and safety of daridorexant between young to middle‐aged adults (aged 18–65 years) and older adults (aged >65 years) (Fietze et al., 2022).

Daridorexant is currently being developed for adult patients with insomnia in Japan. The PK and safety profile of daridorexant in healthy, non‐elderly Japanese and Caucasian subjects have been already shown to be similar (Muehlan, Zuiker, et al., 2020). However, as this study was not conducted in Japan and the aforementioned global Phase 2 and 3 studies did not include any Japanese sites or Japanese patients, a comprehensive clinical evaluation of the safety and efficacy of daridorexant in Japanese subjects has subsequently been conducted. Here we report the results from two early‐phase clinical trials of daridorexant conducted in Japan.

The principal objective of the Phase 1 study was to assess the PK, tolerability, and safety of daridorexant (10, 25, and 50 mg) in healthy Japanese subjects, including older adults. The Phase 2 dose–response study was designed to assess the efficacy and safety of daridorexant at 10, 25, and 50 mg in Japanese adults with insomnia disorder, and to identify the doses to be studied in the Phase 3 programme.

## 2 METHODS

Two separate randomised, double‐blind, placebo‐controlled trials with daridorexant were conducted in Japan between April 2019 and June 2020. The first study was a Phase 1 study conducted in healthy Japanese subjects aged 20–80 years. The second study was a Phase 2 dose‐finding study that enrolled Japanese subjects with insomnia disorder aged 16–65 years. Both studies were conducted in accordance with the International Conference on Harmonisation guidelines for Good Clinical Practice and principles of the Declaration of Helsinki and applicable Japanese laws and regulations. Local Institutional Review Boards reviewed and approved the study protocols plus amendments. All subjects gave written informed consent to participate in this study. The studies were funded by Nxera Pharma Japan Co., Ltd and Mochida Pharmaceutical Co., Ltd.

### 2.1 Phase 1 study

#### 2.1.1 Subjects

The Phase 1 study was conducted at SOUSEIKAI Fukuoka Mirai Hospital Clinical Research Center, Fukuoka City, Fukuoka Prefecture, Japan. The study enrolled healthy subjects (24 subjects aged 20–50 years termed “younger adults” and 24 subjects aged 65–80 years termed “older adults”) with regular sleep patterns of at least 6 h of nocturnal sleep and no clinically significant findings (i.e., no abnormalities in vital signs, electrocardiogram [ECG], or laboratory tests) at the screening physical examinations. The main exclusion criteria included a history or clinical evidence of any diseases (including insomnia disorder) and/or existence of any surgical or medical conditions which might affect the PK of daridorexant, current smoking, caffeine consumption ≥800 mg per day, or any medication ongoing within 2 weeks prior to the first administration of the study drug. Females of childbearing potential were required to have a negative pregnancy test at the screening and on day −1 and to use a reliable method of contraception from the screening until at least 7 days after the last study drug intake. The eligibility criteria were similar to those used for the overseas Phase 1 programme (Muehlan et al., 2018; Muehlan et al., 2019; Muehlan, Zuiker, et al., 2020).

#### 2.1.2 Study design and treatments

This was a double‐blind, randomised, placebo‐controlled, parallel‐group study. Following a screening period of 3 weeks, subjects were randomised to four different daily treatments (placebo, 10, 25, and 50 mg daridorexant tablets) for 4 days, administered in the morning. The study drug was taken with 240 mL water in a fasted state and in a sitting position. No formal statistical hypothesis was made and the sample size of 12 subjects for each dose (six younger and six older adults) was set based on empirical considerations and feasibility. PK profiles were assessed on days 1 and 4 (at steady state). The study design, including the PK sampling schedule, is presented in Figure S1.

#### 2.1.3 Blood sampling and bioanalysis

Serial blood samples for the measurement of daridorexant were collected on day 1 predose until 24 h postdose (=predose day 2), predose on day 3 and 4, and from predose until 72 h post dose on last day of study drug administration on day 4. Details of the PK sample handling and processing including the bioanalytical method have been published previously (Muehlan et al., 2018).

#### 2.1.4 Pharmacokinetic assessments

For PK analysis, concentrations below the limit of quantification were set to zero. The PK variables, including maximum plasma concentration (*C*_max_), time to reach *C*_max_ (*t*_max_), the exposure during a dose interval, that is, area under the plasma concentration–time curve from zero to 24 h (AUC_0–24_), and terminal half‐life (*t*_1/2_), were determined on days 1 and 4 by non‐compartmental analysis using Phoenix WinNonlin (version 8.0; Pharsight Inc., Princeton, NJ, USA). On day 4, the AUC from zero to infinity (AUC_0–∞_), dose proportionality, and accumulation index based on AUC_0–24_ (day 4/day 1) were determined. The PK variables were summarised using descriptive statistics.

#### 2.1.5 Tolerability and safety

Tolerability and safety parameters included change from baseline in vital signs, body weight, laboratory parameters, and 12‐lead ECG, physical examination, and recording of adverse events (AEs). Safety parameters were analysed descriptively using the all‐treated set (all randomised subjects who received at least one dose of study drug).

### 2.2 Phase 2 study

#### 2.2.1 Subjects

The Phase 2 study was conducted at 27 sites in Japan. The eligibility criteria were almost the same as those used for the overseas Phase 2 study (Mignot et al., 2022). Eligible subjects were aged 16–65 years with a diagnosis of insomnia disorder according to the Diagnostic and Statistical Manual of Mental Disorders, 5th Edition (American Psychiatric Association, 2013) and an Insomnia Severity Index (ISI) score ≥15. Subjects were required to have a self‐reported history of disturbed sleep on ≥3 nights per week for ≥3 months prior to screening: ≥30 min to fall asleep, ≥30 min wake time after sleep onset, and a total sleep time ≤6.5 h. During the screening, the same subjective levels of latency to fall asleep, wake time after sleep onset and total sleep time on ≥3 of 7 consecutive nights had to be confirmed with a sleep diary completed daily while polysomnography (PSG) was recorded during two consecutive nights in a single‐blind placebo run‐in period. Additionally, subjects had to meet the following PSG criteria for randomisation: mean latency to persistent sleep (LPS) ≥20 min, mean wake after sleep onset (WASO) ≥30 min and mean total sleep time (TST) <420 min.

Exclusion criteria included any sleep disorder other than insomnia, self‐reported daytime napping (≥1 hour per day on ≥3 days per week), or treatment with a central nervous system‐acting drugs. Subjects with suicidal behaviour or ideation, alcohol or substance abuse, unstable medical conditions, or serious medical disorder were excluded. A full list of inclusion and exclusion criteria is provided in the Supplement.

#### 2.2.2 Study design and treatments

**FIGURE 1:**
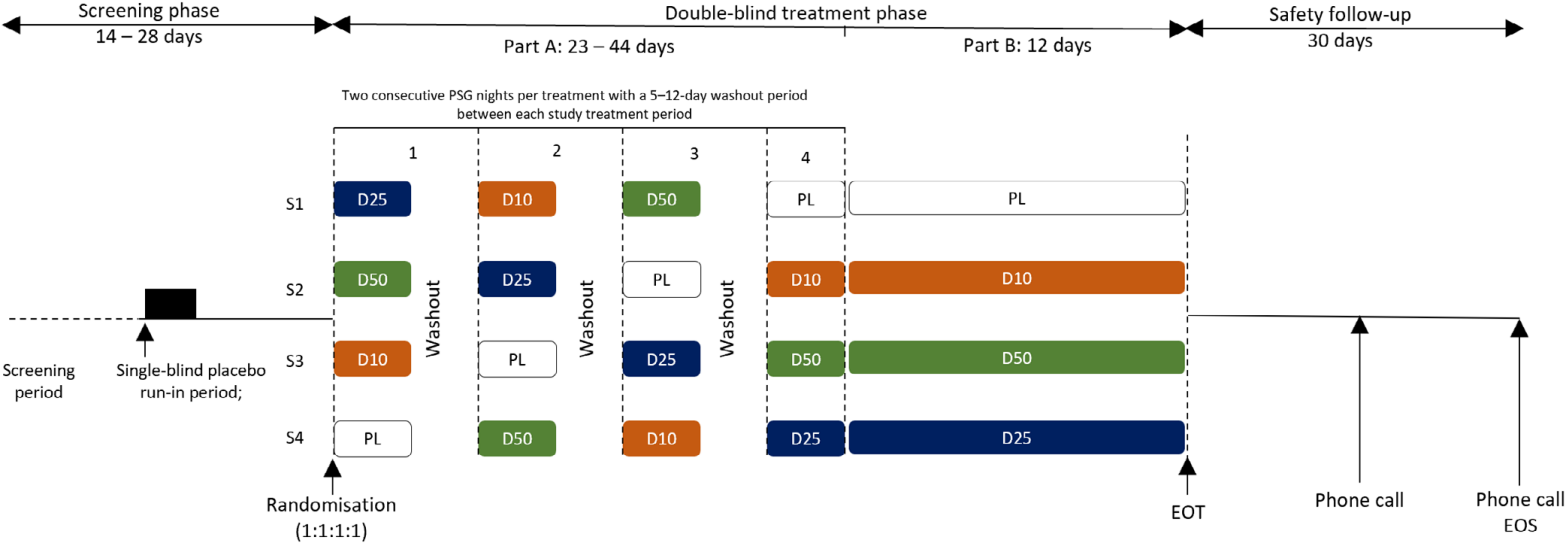
Phase 2 study design. The study consisted of three consecutive phases (screening, double‐blind treatment [Part A and B], safety follow‐up). After a screening period, subjects entered a single‐blind placebo run‐in period; during two consecutive nights, one dose of placebo was administered for each night in a single‐blind manner and overnight polysomnography (PSG) measurements implemented. Eligible subjects were randomised (1:1:1:1) to one of four treatment sequences to receive four treatments (double‐blind treatment of daridorexant [*D*] 10, 25, and 50 mg, and placebo [PL]) during four treatment periods. Subjects received each dose of double‐blind treatment for two consecutive PSG treatment nights as per the assigned treatment sequence, separated by a 5–12 day washout period. Subjects continued the fourth treatment for a further 12 days of double‐blind treatment. Subjects were followed up for safety with two phone calls (5 and 30 days) after the end of treatment (EOT). EOS, end of study.

This was a multicentre, double‐blind, randomised, placebo‐controlled, four‐period, four‐way crossover, PSG dose–response study (Japan Registry of Clinical Trials identifier: jRCT2080224596). The study consisted of three consecutive phases (Figure 1). The screening phase (14–28 days) included a single‐blind placebo run‐in period lasting up to 14 days and was followed by a double‐blind treatment phase of two parts (A and B). Eligible subjects were firstly randomised using an interactive web response system (1:1:1:1, Williams Latin square design) to one of four treatment sequences (Part A). Four study treatments were administered to each subject: daridorexant 10, 25, 50 mg, and placebo. Subjects received each dose of double‐blind treatment for two consecutive PSG treatment nights as per the assigned treatment sequence, with each treatment period separated by a 5–12 day washout period. Subjects continued the treatment received in the last period in Part A for a further 12 days (Part B). The day after the last dose in Part B was defined as the end of treatment (EOT) and was followed by a 30 day safety follow‐up period. Patients, investigators, site personnel, monitors, and sponsor were blinded to treatment allocation until end of study.

#### 2.2.3 Procedures

Study treatment (daridorexant and matching placebo) as a single tablet was taken orally every evening at least 2 h after the evening meal. Treatment was taken ~30 min before lights off. PSG was conducted on two consecutive nights of the run‐in period to provide a baseline and during the two treatment nights of each treatment period in Part A (day 1 and 2) to objectively assess sleep parameters: WASO (time [min] spent awake between onset of persistent sleep and lights on), LPS (time [min] from start of PSG recording [i.e., lights off] to the beginning of the first consecutive 20 non‐wakefulness epochs) and TST (actual sleep time [min]). PSG recordings were centrally evaluated, and all data averaged over the two consecutive recording nights.

Self‐administered sleep diaries were completed at home for at least 7 consecutive days before the run‐in PSG and before EOT and recordings averaged over the 7 days. Diaries were also completed at the clinic in the morning after each PSG night (values averaged over the 2 days) and in the evening before the second PSG night of each treatment period. Questions on the previous night's sleep were completed in the morning and allowed assessment of the following subjective sleep parameters: sWASO (time [min] spent awake after sleep onset), latency to sleep onset (sLSO; time [min] to fall asleep), and sTST (time [min] of total sleep duration). The diary also included a morning visual analogue scale (VAS; range 0–100 with higher score indicating better outcome) to assess morning sleepiness.

#### 2.2.4 Endpoints

The primary efficacy endpoint was a change from baseline in WASO to days 1/2 of each treatment period, as determined by PSG. Secondary endpoints were change in LPS, sWASO, and sLSO from baseline to days 1/2 of each treatment period and change in sWASO and sLSO from baseline to EOT. Changes from baseline in WASO by quarter of the night, TST, sTST, sleep stages (N1, N2, SWS [slow wave sleep], REM), and PSG‐assessed sleep efficiency (100 × TST/time in bed) were also assessed as exploratory endpoints.

For endpoints assessed in Part A, mean objective sleep data from the two placebo run‐in PSG nights and mean values from the sleep diary entries collected during the run‐in period served as baseline. Days 1/2 refer to the mean value of the corresponding two PSG treatment nights (objective endpoints) or corresponding sleep diary entries (subjective endpoints) for a given treatment period. For endpoints assessed in Part B, baseline and EOT refer to the mean values recorded for 7 consecutive nights before the single‐blind placebo run‐in period and EOT, respectively.

Safety and tolerability were assessed by AE reports and change from baseline in vital signs, physical examination, ECG, and clinical laboratory parameters. AEs were coded with the Medical Dictionary for Regulatory Activities (MedDRA) version 23.0. Predefined AEs of special interest (AESI) were narcolepsy‐like events, and suicidal ideation and/or attempt. AESIs were adjudicated by an independent safety board. Next‐morning residual effects (VAS‐assessed morning sleepiness, Digit Symbol Substitution Test [DSST], the Sheehan Disability Scale [SDS], and the Japanese version of the Karolinska Sleepiness Scale [KSS‐J]) and suicidality (Columbia‐Suicide Severity Rating Scale [C‐SSRS]) were also assessed.

#### 2.2.5 Statistical analyses

Assuming the maximum mean reduction in WASO from baseline to days 1/2 with daridorexant was 25 min greater than with placebo (standard deviation [SD] = 40 min) using predefined dose–response models (a linear model and three *E*_max_ models: ED_50_ = 1.11, 3.75, and 15 mg), and taking into account the four treatment groups, a total sample size of approximately 40 in the per protocol set was determined to provide 80%–88% power (power of 84% when averaged over all dose–response models) to reject the null hypothesis (no dose–response relationship) with a 2‐sided significance level of 5%.

The dose–response relationship of daridorexant was analysed with the multiple comparison procedure‐modelling (MCP‐Mod) method (Bretz et al., 2005; Pinheiro et al., 2006; Pinheiro et al., 2014). The MCP‐Mod approach combined a multiple comparison procedure to compare and assess the efficacy of daridorexant with placebo followed by a modelling step to identify a dose that would be most likely to demonstrate the expected level of efficacy based on primary and secondary endpoints. The relationship between each efficacy evaluation and dose was examined using a predefined linear model and the three *E*_max_ models (as stated above). For each model, the null hypothesis was tested against the alternative hypothesis (existence of a dose–response relationship). A dose–response relationship was considered to have been demonstrated if the adjusted *p*‐value was <0.05 for one or more of the four multiple contrast tests. The statistically significant models were fitted to the least squares (LS) mean estimates and associated variance–covariance generated from a linear mixed‐effects model, with baseline value as a covariate, mixed‐effects for dose group and treatment period, and a random effect for subject. The best‐fit model was selected based on the smallest Akaike information criterion value and used to characterise the dose–response relationship.

Efficacy endpoints were analysed using the full analysis set (all subjects who received at least one dose of double‐blind treatment according to dose allocated). Subgroup analyses (sex, age [based on the median age of study population: <52, ≥52 years]; ISI category ≤14, 15–21, ≥22) were performed on the primary endpoint and results summarised descriptively. The safety set included all randomised subjects who received at least one dose of study drug evaluated according to dose actually received. Exploratory and safety endpoints were summarised descriptively. The *p* values presented for analyses other than the MCP‐Mod are nominal without multiplicity adjustment. All statistical analyses were performed using SAS software (version 9.4).

## 3 RESULTS

### 3.1 Phase 1 study

#### 3.1.1 Subjects

In total, 48 healthy Japanese subjects were randomised to placebo or daridorexant 10, 25, and 50 mg and were administered the study drug at least once. Demographic variables were similar across dose groups (Table S1) and the mean (range) age was 26.9 (20–45) years in younger adults and 69.7 (65–78) years in the older adults. One older adult discontinued study drug administration on day 2 due to an AE (mild vertigo positional, judged as related to study drug administration). Since the discontinued subject was not replaced, 47 subjects completed the study.

#### 3.1.2 Pharmacokinetics

**FIGURE 2:**
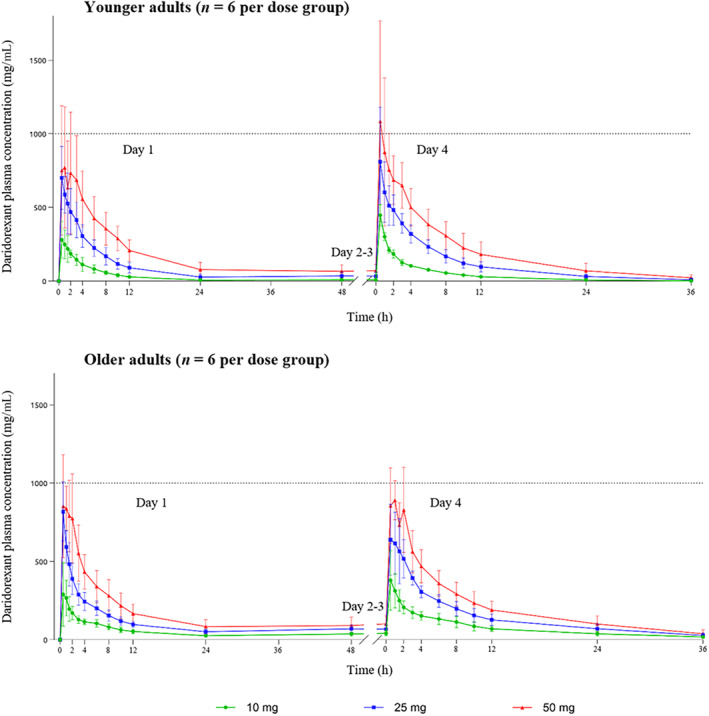
Daridorexant plasma concentration–time profiles (mean, SD) in Japanese younger and older adult subjects on day 1 and day 4 following administration of 10, 25, and 50 mg once daily for 4 days (Phase 1 study). Data are presented as arithmetic mean and standard deviation (SD). Predose samples were taken on days 1, 2, 3, and 4. *N* = 5 for older adult subjects at 25 mg on day 4.

The mean concentration–time profiles in Japanese young adults compared with older adults following administration of daridorexant 10, 25, and 50 mg are depicted in Figure 2, and the PK parameters are summarised in Table 1, while the PK data in the overall population are presented in Table S2. In both age groups, following the study drug administration on day 1 and day 4, daridorexant plasma concentrations increased quickly with a median *t*_max_ of 1.0 h across dose groups. Exposure parameters including *C*_max_ and AUC increased dose‐dependently on both days with minimal to no accumulation (based on AUC_0–24_ day 4/day 1). In the overall population, daridorexant was cleared from plasma with a *t*_1/2_ of 7.42–8.23 h (Table S2). The only notable age‐related difference in PK was the longer *t*_1/2_ of 8.71–11.33 h in older adults compared with 5.43–6.78 h in younger adults (Table 1). Overall, the concentration–time profiles and PK parameters were similar between Japanese younger and older adults.

**TABLE 1:** Summary of main pharmacokinetic parameters in Japanese younger and older adult subjects on day 1 and day 4 following administration of daridorexant 10, 25, and 50 mg once daily for 4 days.

		Daridorexant dose		
PK parameter	Timepoint	10 mg	25 mg	50 mg
Younger adult subjects		*n* = 6	*n* = 6	*n* = 6
*t*_max_ (h)	Day 1	0.75	0.50	0.75
		[0.5, 3.0]	[0.5, 3.0]	[0.5, 3.0]
	Day 4	0.50	0.50	0.50
		[0.5, 0.5]	[0.5, 2.0]	[0.5, 3.0]
*C*_max_ (ng/mL)	Day 1	308.25	720.10	842.39
		(243.71, 389.90)	(567.51, 913.72)	(453.47, 1564.87)
	Day 4	442.43	833.79	1161.78
		(372.46, 525.55)	(597.83, 1162.89)	(725.59, 1860.20)
AUC_0–24_ (h·ng/mL)	Day 1	1407.95	3820.61	6773.43
		(1223.71, 1619.94)	(2940.78, 4963.65)	(4518.63, 10153.38)
	Day 4	1489.32	4030.45	6525.32
		(1333.64, 1663.18)	(3180.54, 5107.48)	(4645.20, 9166.39)
*t*_1/2_ (h)	Day 1	5.43	5.97	6.78
		(4.29, 6.88)	(4.54, 7.85)	(5.42, 8.49)
	Day 4	5.73	6.33	6.33
		(4.87, 6.74)	(5.24, 7.64)	(4.52, 8.86)
AI	Day 4/Day 1	1.06	1.06	0.97
		[0.9, 1.2]	[0.9, 1.3]	[0.7, 1.2]
Older adult subjects		*n* = 6	*n* = 6	*n* = 6
*t*_max_ (h)	Day 1	1.00	0.50	0.75
		[0.5, 2.0]	[0.5, 1.0]	[0.5, 2.0]
	Day 4	0.50	1.00	1.00
		[0.5, 1.5]	[0.5, 2.0]	[0.5, 2.0]
*C*_max_ (ng/mL)	Day 1	306.66	807.50	1046.57
		(177.89, 528.64)	(638.70, 1020.93)	(878.94, 1246.16)
	Day 4	376.93	654.61	1021.54
		(231.09, 614.80)	(407.37, 1051.89)	(852.66, 1223.87)
AUC_0–24_ (h·ng/mL)	Day 1	1785.94	3753.56	6148.17
		(1408.66, 2264.25)	(3025.95, 4656.13)	(4601.64, 8214.45)
	Day 4	2376.38	4598.69	6705.32
		(1889.59, 2988.56)	(3576.26, 5913.44)	(5257.83, 8551.30)
*t*_1/2_ (h)	Day 1	10.90	11.33	9.55
		(9.69, 12.28)	(8.56, 14.99)	(7.65, 11.93)
	Day 4	10.83	9.60	8.71
		(8.84, 13.27)	(7.24, 12.73)	(6.94, 10.94)
AI	Day 4/Day 1	1.34	1.16	1.10
		[1.1, 1.5]	[1.0, 1.3]	[1.0, 1.3]

*Note*: Data are expressed as geometric mean (95% confidence interval) except for the accumulation index (arithmetic mean[range]) and for *t*_max_ which is expressed as median [range]. *N* = 5 for older adults at 25 mg on day 4.

Abbreviations: AI, accumulation index (based on AUC_0–24_); AUC_0–24_, area under the plasma concentration–time curve from time 0 to 24 h; *C*_max_, maximum plasma concentration; PK, pharmacokinetic; *t*_1/2_, terminal half‐life; *t*_max_, time to reach *C*_max_.

#### 3.1.3 Tolerability and safety

A summary of all treatment‐emergent AEs is shown in Table S3. All AEs reported were mild in severity and no deaths or serious AEs occurred during the study. One AE of mild vertigo positional led to discontinuation of study drug in an older adult on day 2 receiving daridorexant 25 mg. The most frequently reported AE after morning administration of daridorexant was somnolence, reported by 50.0%, 91.7%, 91.7%, and 16.7% of subjects receiving daridorexant 10, 25, 50, and placebo, respectively (Table S3). No treatment‐emergent clinically relevant abnormalities of vital signs, physical examination, 12‐lead ECG recordings, or clinical laboratory variables were detected at any dose.

### 3.2 Phase 2 study

#### 3.2.1 Subjects

A total of 118 subjects were screened, of which 47 were randomised to one of the four treatment sequences (Figure S2). All 47 subjects received all four assigned treatments, progressed to Part B and completed the study. During Part B, the number of subjects in each treatment group was 11 or 12 as the design of the second study part was not a crossover. Demographic and baseline characteristics were generally balanced between each randomised sequence group with the exception of time since diagnosis (Table 2). At the screening, 53% of subjects were female, the mean (range) age was 50.4 (26–64) years, and the mean (SD) ISI was 19.4 (3.11).

**TABLE 2:** Subject demographics and baseline characteristics of the Phase 2 study.

Characteristic	Randomised treatment sequence, mg				Total subjects
	1	2	3	4	
	25‐10‐50‐P	50‐25‐P‐10	10‐P‐25‐50	P‐50‐10‐25	
	(*n* = 12)	(n = 12)	(*n* = 11)	(*n* = 12)	(*n* = 47)
Sex, *n* (%)					
Female	6 (50)	8 (67)	6 (55)	5 (42)	25 (53)
Male	6 (50)	4 (33)	5 (45)	7 (58)	22 (47)
Mean age, years (range)	50.4 (39, 59)	49.7 (39, 62)	48.1 (26, 59)	53.1 (31, 64)	50.4 (26, 64)
<52 years, *n* (%)	5 (42)	7 (58)	6 (55)	4 (33)	22 (47)
≥52 years, *n* (%)	7 (58)	5 (42)	5 (45)	8 (67)	25 (53)
Mean BMI, kg/m^2^ (SD)	22.4 (2.53)	21.4 (2.02)	23.9 (2.57)	23.4 (2.32)	22.7 (2.48)
Mean ISI (SD)	19.7 (2.67)	19.2 (3.38)	19.0 (3.03)	19.9 (3.60)	19.4 (3.11)
Mean time since insomnia diagnosis, years (SD)	9.9 (12.4)	5.6 (5.7)	5.9 (5.0)	11.3 (7.0)	8.2 (8.3)

Abbreviations: BMI, body mass index; ISI, insomnia severity index; P, placebo; SD, standard deviation.

#### 3.2.2 Primary and secondary efficacy endpoints

**FIGURE 3:**
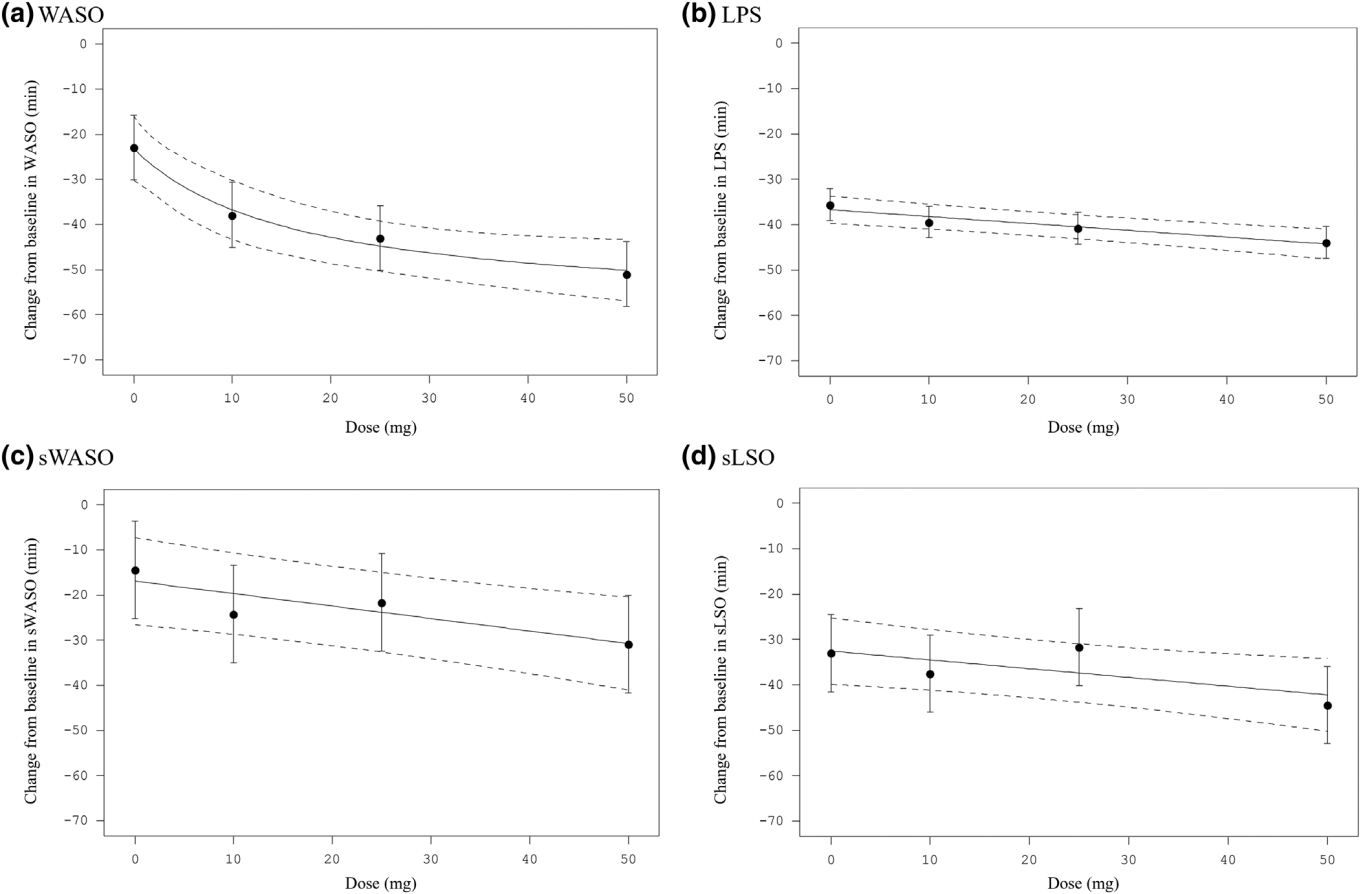
Predicted dose–response profile and least squares (LS) means for change in WASO (primary endpoint) and LPS, sWASO and sLSO (secondary endpoints) from baseline to days 1/2 (Phase 2 study). The predicted mean (solid line; 95% confidence interval [CI] — dashed line) dose–response relationship for change from baseline to days 1/2 in wake after sleep onset (WASO), latency to persistent sleep (LPS) and subjective WASO (sWASO) and latency to sleep onset (sLSO) over the dose range in the Phase 2 study, using the *E*_max_ dose–response model (WASO) and linear dose–response model (LPS, sWASO, sLSO). Points and bars represent LS means and 95% CI, respectively.

A statistically significant (*p* < 0.0001, MCP‐Mod method; *E*_max_ model) dose–response relationship in the reduction of PSG‐assessed WASO (primary endpoint) from baseline to days 1/2 was observed for daridorexant (Figure 3). The LS mean estimates of the change from baseline to days 1/2 were statistically significant for all doses of daridorexant compared with placebo (Table 3). The dose‐dependent decrease in WASO from baseline to days 1/2 was observed in all four quarters of the 8‐h night and the effect increased as the night progressed (Table S4), and across the prespecified subgroups (between adults and older adults, between males and females, and across ISI categories) (Table S5).

**TABLE 3:** Absolute values and change from baseline to day 1/2 in objective (WASO, LPS, TST) and subjective (sWASO, sLSO, sTST) sleep parameters (Phase 2 study).

Endpoint		Placebo	Daridorexant dose		
			10 mg	25 mg	50 mg
		(*n* = 47)	(*n* = 47)	(*n* = 47)	(*n* = 47)
Primary endpoint					
WASO, min	Baseline	84.2 (40.5)	84.2 (40.5)	84.2 (40.5)	84.2 (40.5)
	Days 1/2	61.2 (34.1)	46.2 (23.7)	41.2 (24.4)	33.3 (29.7)
	LS mean change (95% CI)	−22.9 (−30.1, −15.7)	−37.9 (−45.1, −30.7)	−43.0 (−50.2, −35.8)	−51.0 (−58.2, −43.8)
	Placebo‐corrected LS mean (95% CI)	‐	−14.9 (−23.1, −6.7)	−20.1 (−28.3, −11.9)	−28.0 (−36.2, −19.9)
	*p* value	‐	0.0004	<0.0001	<0.0001
Secondary endpoints					
LPS, min	Baseline	57.0 (47.7)	57.0 (47.7)	57.0 (47.7)	57.0 (47.7)
	Days 1/2	21.4 (17.3)	17.5 (12.3)	16.2 (11.3)	13.1 (9.5)
	LS mean change (95% CI)	−35.6 (−39.1, −32.1)	−39.4 (−42.9, −35.9)	−40.8 (−44.3, −37.2)	−43.9 (−47.4, −40.4)
	Placebo‐corrected LS mean (95% CI)	‐	−3.8 (−7.6, 0.0)	−5.1 (−8.9, −1.4)	−8.3 (−12.0, −4.5)
	*p* value	‐	0.0476	0.0078	<0.0001
sWASO, min	Baseline	93.5 (49.5)	93.5 (49.5)	93.5 (49.5)	93.5 (49.5)
	Days 1/2	78.9 (45.7)	69.1 (38.4)	71.9 (49.4)	62.7 (47.9)
	LS mean change (95% CI)	−14.4 (−25.3, −3.6)	−24.2 (−35.0, −13.4)	−21.7 (−32.5, −10.9)	−30.9 (−41.7, −20.1)
	Placebo‐corrected LS mean (95% CI)	‐	−9.7 (−19.8, 0.3)	−7.2 (−17.3, 2.8)	−16.5 (−26.5, −6.5)
	*p* value	‐	0.0561	0.1544	0.0014
sLSO, min	Baseline	73.6 (47.2)	73.6 (47.2)	73.6 (47.2)	73.6 (47.2)
	Days 1/2	40.6 (27.4)	35.9 (30.0)	42.0 (47.0)	29.3 (30.0)
	LS mean change (95% CI)	−33.0 (−41.5, −24.5)	−37.5 (−46.0, −29.0)	−31.7 (−40.2, −23.2)	−44.5 (−53.0, −36.0)
	Placebo‐corrected LS mean (95% CI)	‐	−4.5 (−13.5, 4.5)	1.4 (−7.6, 10.3)	−11.4 (−20.4, −2.5)
	*p* value	‐	0.3246	0.7616	0.0128
Exploratory endpoints					
TST, min	Baseline	349.6 (53.9)	349.6 (53.9)	349.6 (53.9)	349.6 (53.9)
	Days 1/2	402.4 (39.8)	420.3 (26.5)	426.3 (26.9)	437.3 (32.1)
	LS mean change (95% CI)	52.8 (44.8, 60.8)	70.6 (62.6, 78.5)	76.7 (68.7, 84,6)	87.7 (79.8, 95.7)
	Placebo‐corrected LS mean (95% CI)	‐	17.8 (8.6, 26.9)	23.9 (14.7, 33.0)	34.9 (25.8, 44.1)
	*p* value		0.0002	<0.0001	<0.0001
sTST, min	Baseline	313.2 (68.5)	313.2 (68.5)	313.2 (68.5)	313.2 (68.5)
	Days 1/2	360.5 (61.8)	375.0 (55.0)	366.2 (80.0)	387.3 (62.5)
	LS mean change (95% CI)	47.2 (32.1, 62.2)	61.4 (46.3, 76.4)	53.0 (37.9, 68.1)	74.4 (59.4, 89.5)
	Placebo‐corrected LS mean (95% CI)	‐	14.2 (−0.6, 29.1)	5.9 (−9.0, 20.7)	27.3 (12.4, 42.2)
	*p* value		0.0607	0.4380	0.0004

*Note*: Data are presented as mean (SD) unless otherwise stated. Absolute values and changes (least squares [LS] mean) from baseline to days 1/2 in wake after sleep onset (WASO; primary endpoint), latency to persistent sleep (LPS; secondary endpoint), subjective WASO (sWASO; secondary endpoint), subjective latency to sleep onset (sLSO; secondary endpoint), total sleep time (TST) and self‐reported TST (sTST) in patients with insomnia disorder receiving daridorexant 10, 25, 50 mg or placebo. For polysomnography (PSG) measures, baseline is the mean of the two overnight PSG measurements during the run‐in period and days 1/2 is the mean value of the corresponding 2 overnight PSG measurements. For subjective measures, baseline is the mean value of the sleep diary entries during the two consecutive days in the run‐in period and days 1/2 is the mean value of the two corresponding sleep diary entries in each double‐blind treatment period. As all patients received all four treatments, the baseline (from run‐in period) is the same across the treatment groups. LS mean calculated with a linear mixed‐effects model (change from baseline = dose group + period + baseline + subject [random]).

Abbreviations: EOT, end of treatment; LPS, latency to persistent sleep; LSO, latency to sleep onset; sLSO, subjective latency to sleep onset; sTST, self‐reported total sleep time; sWASO, subjective wake after sleep onset; TST, total sleep time; WASO, wake after sleep onset.

A statistically significant (*p* < 0.0001, MCP‐Mod method, linear model) dose–response relationship in the reduction of LPS (secondary endpoint) from baseline to days 1/2 was also observed for daridorexant (Figure 3). Again, the LS mean estimates of the change from baseline to days 1/2 in LPS were statistically significant for all doses of daridorexant compared with placebo (Table 3).

For the subjective secondary endpoints, statistically significant dose‐dependent reductions in sWASO and sLSO (MCP‐Mod method, linear model, *p* = 0.0084 and 0.0457 respectively) from baseline to days 1/2 were observed for daridorexant (Figure 3). Reductions were significantly greater for daridorexant 50 mg versus placebo (Table 3). Numerically greater reductions from baseline in sWASO and sLSO were observed at EOT with daridorexant 50 mg compared with placebo; differences were not statistically significant (Table 4).

**TABLE 4:** Absolute values and changes from baseline to end of treatment (EOT) in subjective sleep parameters (sWASO, sLSO, sTST) (Phase 2 study).

Endpoint		Placebo	Daridorexant dose		
			10 mg	25 mg	50 mg
		(*n* = 12)	(*n* = 12)	(*n* = 12)	(*n* = 11)
sWASO, min	Baseline	96.5 (38.2)	113.8 (67.3)	78.1 (39.0)	84.8 (46.5)
	EOT	48.9 (31.4)	47.7 (33.7)	51.8 (29.2)	34.9 (37.5)
	LS mean change (95% CI)	−45.1 (−63.9, −26.3)	−49.2 (−68.4, −30.0)	−39.0 (−58.0, −20.0)	−57.1 (−76.7, −37.4)
	Placebo‐corrected LS mean (95% CI)	‐	−4.1 (−30.9, 22.7)	6.1 (−20.7, 32.9)	−12.0 (−39.2, 15.3)
	*p* value	‐	0.7573	0.6495	0.3802
sLSO, min	Baseline	75.6 (59.4)	76.0 (47.2)	62.3 (28.8)	81.1 (52.7)
	EOT	44.4 (51.4)	53.2 (43.0)	36.7 (16.9)	21.9 (14.5)
	LS mean change (95% CI)	−29.9 (−48.7, −11.1)	−21.3 (−40.1, −2.4)	−33.2 (−52.1, −14.2)	−54.2 (−73.9, −34.5)
	Placebo‐corrected LS mean (95% CI)	‐	8.6 (−18.0, 35.3)	−3.3 (−30.1, 23.5)	−24.3 (−51.6, 2.9)
	*p* value	‐	0.5163	0.8058	0.0790
sTST, min	Baseline	307.9 (81.6)	290.2 (70.6)	339.6 (40.5)	315.5 (74.0)
	EOT	378.6 (60.3)	360.6 (53.8)	390.9 (38.1)	428.4 (65.7)
	LS mean change (95% CI)	66.7 (35.6, 97.7)	53.0 (21.5, 84.5)	71.2 (39.6, 102.8)	114.6 (82.3, 147.0)
	Placebo‐corrected LS mean (95% CI)		−13.7 (−57.7, 30.4)	4.6 (−39.9, 49.0)	48.0 (3.1, 92.8)
	*p* value	‐	0.5343	0.8373	0.0366

*Note*: Data are presented as mean (SD) unless otherwise stated. Absolute values and changes (least squares [LS] mean) from baseline to end of treatment (EOT) in subjective wake after sleep onset (sWASO, secondary endpoint), latency to sleep onset (sLSO, secondary endpoint) and self‐reported total sleep time (sTST; exploratory) in patients with insomnia disorder receiving daridorexant 10, 25, 50 mg or placebo. Baseline and EOT are the mean values recorded for 7 consecutive nights before the single‐blind placebo run‐in period and EOT. LS mean calculated with a linear model (change from baseline = dose group + baseline).

Abbreviations: EOT, end of treatment; sLSO, subjective latency to sleep onset; sTST, self‐reported total sleep time; sWASO, subjective wake after sleep onset.

#### 3.2.3 Other efficacy endpoints

No adjustments for multiplicity were performed for the exploratory endpoints. A dose‐dependent increase in PSG‐measured TST (*p* < 0.0001; *E*_max_ model) and sTST (*p* = 0.0032, linear model) from baseline to days 1/2 was observed with daridorexant. The LS mean estimates of the change from baseline to days 1/2 in TST were greater for all doses of daridorexant compared with placebo (Table 3; all *p* < 0.05). For sTST, the LS mean estimates were greater for daridorexant 50 mg versus placebo (*p* = 0.0004). Mean changes from baseline in sTST at EOT were also larger for daridorexant 50 mg versus placebo at EOT (*p* = 0.0366; Table 4). The proportion of TST spent in REM sleep increased with increasing dose of daridorexant from baseline to days 1/2 (Table S6). The increases in TST translated into increased PSG sleep efficiency which improved in a dose‐dependent manner from baseline (72.8%) to days 1/2 (91.1% with daridorexant 50 mg; Table S7).

#### 3.2.4 Safety

The overall incidence of TEAEs in Part A and B was higher in the daridorexant treatment groups compared with placebo but all AEs were mild or moderate in severity and did not lead to any treatment discontinuation (Table 5). No deaths or serious AEs were reported throughout the study. The most common TEAE in both parts of the study was somnolence; in Part A, somnolence was reported in 1, 3, 2, and 4 subjects in the placebo, 10, 25, and 50 mg groups respectively; in Part B, somnolence was reported in 1, 1, 0 and 1 subjects in the placebo, 10, 25, and 50 mg respectively. Nightmares were also reported in two subjects receiving daridorexant 50 mg in Part A. Independently adjudicated AESIs, of mild intensity, were reported in three subjects (all in Part B). Events associated with excessive daytime sleepiness (somnolence) were reported in two subjects (daridorexant 10 mg [*n* = 1], daridorexant 50 mg [*n* = 1]), and one patient receiving daridorexant 50 mg reported sleep paralysis. No events denoting other complex sleep behaviours were reported. VAS scores for morning sleepiness showed a numerical increase (i.e., improvement) from baseline to EOT in all treatment groups, with the biggest improvements recorded for daridorexant 25 and 50 mg (Table 5).

**TABLE 5:** Summary of treatment‐emergent adverse events and morning sleepiness based on visual analogue scale (VAS) (Phase 2 study).

TEAEs	Placebo	Daridorexant dose		
		10 mg	25 mg	50 mg
Part A				
*N*	47	47	47	47
Participants with ≥1 TEAE, *n* (%)	3 (6.4)	5 (10.6)	4 (8.5)	7 (14.9)
TEAE occurring in ≥2 subjects in any group, *n* (%)				
Somnolence	1 (2.1)	3 (6.4)	2 (4.3)	4 (8.5)
Headache	0	2 (4.3)	0	0
Nightmare	0	0	0	2 (4.3)
Part B				
*N*	12	12	12	11
Participants with ≥1 TEAE, *n* (%)	1 (8.3)	2 (16.7)	2 (16.7)	3 (27.3)
TEAE occurring in ≥1 subjects in any group, *n* (%)	0	0	0	0
Somnolence	1 (8.3)	1 (8.3)	0	1 (9.1)
Sleep attacks	0	0	0	1 (9.1)
Malaise	0	0	0	1 (9.1)
Ligament sprain	0	0	0	1 (9.1)
Muscle spasms	0	0	0	1 (9.1)
Abnormal hepatic function	0	0	1 (8.3)	0
Viral gastroenteritis	0	0	1 (8.3)	0
Nasopharyngitis	0	1 (8.3)	0	0
AESI after ISB adjudication, *n* (%)	0	1 (8.3)	0	2 (18.2)
Somnolence	0	1 (8.3)	0	1 (9.1)
Complex sleep behaviour	0	0	0	1 (9.1)
Morning sleepiness, mm^a^, mean (SD)				
Baseline	44.0 (21.8)	45.9 (25.7)	36.2 (15.6)	39.6 (15.6)
EOT	59.1 (21.3)	59.0 (26.1)	54.3 (13.9)	57.9 (21.3)
Change to EOT^b^	15.1 (15.8)	13.1 (31.2)	18.1 (13.3)	18.2 (26.1)

Abbreviations: AESI, adverse event of special interest; EOT, end of treatment; ISB, independent safety board; SB, single‐blind; TEAE, treatment‐emergent adverse event; VAS, visual analogue scale.

^a^ Visual analogue scale (VAS) score ranges from 0 to 100; from 0 “very sleepy” to 100 “not sleepy at all”. A higher score indicates less morning sleepiness.

^b^ An increase in VAS morning sleepiness score from baseline indicates an improvement in morning sleepiness.

Scores for DSST (attention, perception speed, motion speed, visual scanning, memory), KSS‐J (next‐day alertness) and SDS (impairment in work, social life, and home responsibilities) evaluations were conducted on the morning of the day after administration during the double‐blind treatment period of Part A and at EOT; no notable effect of daridorexant, or dose‐dependent change was observed in any of these evaluations (Table S8). Per the C‐SSRS, no suicidal ideation or behaviour was reported during the study. No clinically relevant changes in laboratory variables, vital signs, ECG, or body weight were observed.

## 4 DISCUSSION

These are the first two studies conducted in Japan to evaluate the PK, safety, and efficacy of daridorexant. Overall, the results from these early‐phase clinical trials are consistent with those observed in studies previously conducted in non‐Japanese individuals, in both healthy adults and adults with insomnia disorder, including older adults (Dauvilliers et al., 2020; Fietze et al., 2022; Kunz et al., 2023; Mignot et al., 2022; Muehlan et al., 2018; Muehlan et al., 2019; Muehlan, Zuiker, et al., 2020; Zammit et al., 2020) and support further investigation of daridorexant in Japanese patients with insomnia disorder in Phase 3.

In healthy Japanese adults and older adults, the PK of daridorexant were consistent with previous results reported in healthy Caucasian and Japanese individuals (Muehlan et al., 2018; Muehlan et al., 2019; Muehlan, Zuiker, et al., 2020). Daridorexant, at a dose of 10, 25, or 50 mg, was rapidly absorbed with a *t*_max_ reached within 1 h. Thereafter, plasma concentrations declined and daridorexant was cleared from plasma with a *t*_1/2_ of ~8 h. Exposure (*C*_max_ and AUC) increased dose‐dependently for all doses evaluated. The PK parameters *t*_max_, *C*_max_, and AUC were not affected by age, whereas the *t*_1/2_ increased from ~6–7 h in younger adults to 9–12 h in older adults. These observed differences between younger adults and older adults are in line with previous observations in the overseas Phase 1 programme (Muehlan et al., 2018; Muehlan et al., 2019) as well as with the well‐documented physiological decrease in intrinsic metabolic drug clearance that occurs with advancing age in the order of 20%–60% (Butler & Begg, 2008; Rowland et al., 2011).

While overall the plasma concentration–time profiles were similar between younger and older adult subjects, the observed tendency for a prolonged elimination in the elderly is in line with previous observations (Muehlan, Zuiker, et al., 2020) and can readily be explained by the reduced intrinsic clearance, that is, reduced CYP3A4 metabolic activity associated with increased age (Cotreau et al., 2005; Rowland et al., 2011). The prolonged elimination of daridorexant and the associated slightly increased residual next‐morning concentrations in older adults did not lead to next‐day residual drug effects in the subjects with insomnia as studied in the overseas Phase 2 and 3 studies (Mignot et al., 2022; Zammit et al., 2020). This should be discussed in further Japanese Phase 3 studies. Notably, upon repeated dosing, no clinically relevant accumulation was observed in Japanese subjects in the present study, which is in line with previous results in Caucasian subjects (Muehlan et al., 2019). Administration of daridorexant was safe and well tolerated in healthy Japanese subjects, all treatment‐emergent AEs were of mild intensity. The most frequently reported AE was somnolence, which was expected because the insomnia medication was administered in the morning to individuals without insomnia disorder. In the Phase 2 study, where daridorexant was administered in the evening to patients with insomnia, somnolence was reported at much lower rates in all groups (Parts A and B).

In line with the observed PK profile in healthy Japanese subjects and similar to the overseas Phase 2 and 3 studies (Dauvilliers et al., 2020; Mignot et al., 2022; Zammit et al., 2020), in Japanese patients with insomnia disorder, short‐term treatment over two nights with daridorexant significantly improved, in a dose‐dependent manner, sleep maintenance (WASO) and sleep induction (LPS), as assessed objectively by PSG. All doses of daridorexant studied showed a statistically significant improvement compared with placebo for both endpoints. In addition to the effects on WASO and LPS, TST, an exploratory endpoint, also increased in a dose‐dependent manner. In particular, we observed a numerical increase in the proportion of TST spent in REM sleep with increasing dose. The dose–response observed for WASO was consistent between adults and older adults, between males and females, and across ISI categories. When assessing WASO across the different quarters of the night, the results of this study show that daridorexant decreases wakefulness along the entire 8 h night and the effect increases as the night progresses. These results are again in agreement with what has been observed in non‐Japanese patients (Di Marco et al., 2023). The objective PSG endpoints are supported by the self‐reported correlates (sWASO, sLSO, sTST), which also showed the dose‐dependent efficacy of daridorexant on days 1/2, with the 50 mg dose showing statistically significant improvements compared with placebo on all three efficacy endpoints. A significant improvement versus placebo in sTST was also observed up to EOT in Part B with daridorexant 50 mg.

The increase in the proportion of REM sleep seen with increasing dose of daridorexant at days 1/2 is an interesting observation. This is in contrast to Z‐drugs which decrease REM sleep (de Mendonça et al., 2023). The clinical implications of increased REM sleep are unclear, but the literature suggests they are likely beneficial and may include improvements in memory and concentration (Goldstein & Walker, 2014; Lau et al., 2015). As REM sleep mostly occurs during the second half of the night, an increase may also indicate improved sleep quality (Barbato, 2021). Although dream content is more likely to be recalled during REM sleep, in the current study, the incidence of nightmares/abnormal dreams were not common events (*n* = 2 daridorexant 50 mg, Part A).

In terms of the safety profile of daridorexant, no new safety signals were found in these two Japanese studies. In the Phase 2 study, no severe AEs, deaths, or AEs leading to discontinuation were reported. One of the most common AEs was somnolence, but the incidence was low in all groups and consistent with previous studies of daridorexant, there were no dose‐limiting safety issues in the dose range studied (Dauvilliers et al., 2020; Mignot et al., 2022; Zammit et al., 2020). Independently adjudicated AESIs were reported in three subjects but were of mild intensity and there were no cases of cataplexy. There was no evidence of suicidal ideation or behaviour. As expected with the PK profile of daridorexant (i.e., the short half‐life of 8 h), there was also no evidence of next‐morning residual drug effects up to the highest dose. The potential for next‐morning residual sedative effects which can interfere with daytime functioning and quality of life is a main concern with insomnia medications (Roth et al., 2011). Daridorexant was developed to target an optimal half‐life long enough to cover the duration of the night but short enough to minimise next‐morning residual effects. Compared with suvorexant (half‐life ~12 h) (U.S Food and Drug Administration, 2014) and lemborexant (effective half‐life 17–19 h) (U.S Food and Drug Administration, 2019), daridorexant has the shortest half‐life (8 h) of the three DORAs. Accumulation resulting from a longer half‐life of >10 h may carry a risk of residual hangover effects due to increased plasma concentrations the next morning (Muehlan, Vaillant, et al., 2020). In all groups, including placebo, the DSST score increased and KSS‐J and SDS scores decreased from baseline to days 1/2 and to EOT, suggesting no negative effect on cognitive function, daytime sleepiness or disability the next morning. The data also indicate improvements in next‐morning sleepiness (VAS) with daridorexant 25 and 50 mg compared with placebo, in accordance with observations in the overseas studies (Kunz et al., 2023; Mignot et al., 2022).

We do acknowledge that a limitation of the Phase 1 study is that, compared with the parallel design used in this study, a within‐subject control (crossover design) would be more robust and potentially reduce variability in the evaluation of the PK. In addition, in the Phase 2 study, the study design in Part B lessened the ability to detect significant changes due to the small sample size per group. Studies with a larger sample size with a longer duration are required to further assess the efficacy and safety of daridorexant in Japanese patients.

In conclusion, these two trials investigated daridorexant in Japanese subjects (and for the first time in Japanese patients with insomnia disorder), and the data are consistent with previous observations in the overseas development programme. Daridorexant was quickly absorbed and cleared from plasma with a *t*_1/2_ of ~8 h and resulted in a significant dose–response (i.e., improvement) of objective and subjective sleep onset and sleep maintenance variables in Japanese subjects with insomnia disorder, without any dose‐limiting safety issues. The dose‐dependent improvement in sleep maintenance (WASO) was observed across the full 8 h night, with the effect increasing as the night progressed. These results suggest that daridorexant could become a treatment option for Japanese patients with insomnia disorder. Daridorexant 50 mg, which significantly improved objective and subjective insomnia symptoms compared with placebo, and daridorexant 25 mg which showed higher efficacy than 10 mg for the objective endpoints of sleep onset, sleep maintenance and total sleep time, have since been further investigated in Phase 3 trials in Japanese patients with insomnia disorder (Japan Registry of Clinical Trials jRCT2031200452 and jRCT2080225348).

## AUTHOR CONTRIBUTIONS

**Makoto Uchiyama:** Writing – review and editing; conceptualization; investigation; methodology. **Kazuo Mishima:** Writing – review and editing; conceptualization; investigation; methodology. **Tomoko Yagi:** Writing – review and editing; conceptualization; investigation; methodology. **Tatsuya Yoshihara:** Writing – review and editing; conceptualization; investigation; methodology. **Takashi Eto:** Writing – review and editing; conceptualization; investigation; methodology. **Clemens Muehlan:** Writing – original draft; writing – review and editing; conceptualization; validation; formal analysis; methodology. **Osamu Togo:** Writing – review and editing; data curation; formal analysis; validation; investigation; methodology; software. **Yuichi Inoue:** Writing – review and editing; methodology; conceptualization; investigation.

## FUNDING INFORMATION

The studies were funded by Nxera Pharma Japan Co., Ltd and Mochida Pharmaceutical Co., Ltd.

## CONFLICT OF INTEREST STATEMENT

Makoto Uchiyama reports that he has provided consulting services and received personal fees from Nxera Pharma Japan Co., Ltd, Lixil Corporation and Taisho Pharmaceutical Co., Ltd. Kazuo Mishima has received speaker's honoraria from EISAI Co., Ltd., Nobelpharma Co., Ltd, Takeda Pharmaceutical Co., Ltd, MSD Inc. and research grants from Eisai Co., Ltd, Sumitomo Pharma Co., Ltd, Takeda Pharmaceutical Co., Ltd. Tomoko Yagi provided central scoring and received personal fees from Nxera Pharma Japan Co., Ltd. Tatsuya Yoshihara declares no conflict of interest. Takashi Eto declares no conflict of interest. Clemens Muehlan is an employee and shareholder of Idorsia Pharmaceuticals Ltd. Osamu Togo is an employee of Nxera Pharma Japan Co., Ltd. Yuichi Inoue has received personal fees from Eisai Co., Ltd, MSD K.K., Takeda Pharmaceutical Co., Ltd, Astellas Pharma Inc., and grants from Philips Japan Co., Ltd, KOIKE Medical Co., Ltd, TEIJIN LIMITED.

## PATIENT CONSENT STATEMENT

In both studies, all subjects gave written informed consent.

## CLINICAL TRIALS REGISTRATION

Japan Registry of Clinical Trials: jRCT2080224596 (https://jrct.niph.go.jp/latest-detail/jRCT2080224596).

## Supporting information


**Data S1.** Supporting Information.

## Data Availability

The data that support the findings of this study are available on request from the corresponding author. The data are not publicly available due to privacy or ethical restrictions.
