# Supplementary material for: Pharmacokinetics, safety, and efficacy of daridorexant in Japanese subjects: Results from phase 1 and 2 studies
Source: J Sleep Res. 2024 Aug 8;34(1):e14302. doi: 10.1111/jsr.14302 (PMC11744248; doi:10.1111/jsr.14302)
Supplement: Supplementary file 1 — Data S1. Supporting Information. [file JSR-34-e14302-s001.docx]

**Supplementary Material**

**Pharmacokinetics, safety, and efficacy of daridorexant in Japanese subjects: results from Phase 1 and 2 studies**

Makoto Uchiyama^1,2^, Kazuo Mishima^3^, Tomoko Yagi^4^, Tatsuya Yoshihara^5^, Takashi Eto^6^, Clemens Muehlan^7^, Osamu Togo^8^, Yuichi Inoue^9^

^1^Department of Psychiatry, Nihon University School of Medicine, Itabashi, Tokyo, Japan;

^2^Tokyo Adachi Hospital, Adachi, Tokyo, Japan; ^3^Department of Neuropsychiatry, Akita University Graduate School of Medicine, Akita, Japan; ^4^Kurume University School of Medicine, Fukuoka, Japan;

^5^SOUSEIKAI Fukuoka Mirai Hospital Clinical Research Center, Fukuoka, Japan; ^6^SOUSEIKAI Hakata Clinic, Fukuoka, Japan; ^7^Clinical Pharmacology, Idorsia Pharmaceuticals Ltd, Allschwil, Switzerland; ^8^Data Management & Biometry, Nxera Pharma Japan Co., Ltd, Tokyo, Japan;

^9^Yoyogi Sleep Disorder Center, Tokyo, Japan.

**Phase 2 study inclusion and exclusion criteria**

*Inclusion Criteria*

Subjects who met all of the following criteria were included in the study:

1. Provided informed consent
2. Japanese subjects aged 16–65 years
3. Body mass index 18.5-31.9 kg/m^2^ at screening
4. Insomnia disorder based on Diagnostic and Statistical Manual of Mental Disorders, 5th Edition
5. Self-reported history of all of the following for at least 3 months prior to Visit 1 and on at least three nights per week:
   1. ≥30 min to fall asleep
   2. Wake time during sleep ≥30 min
   3. Total sleep time (TST) ≤6.5 h
6. Insomnia Severity Index score ≥15
7. The following sleep parameters for three nights or more, according to the sleep diary completed at home for seven consecutive nights between Visit 1 and Visit 2:
   1. ≥30 min to fall asleep
   2. Wake time during sleep ≥30 min
   3. TST ≤6.5 h
8. Subjects with 6–9 h of normal time in bed, as reported by the sleep diary completed at home between Visit 1 and Visit 2
9. Sleep parameters meeting the following criteria confirmed by two overnight polysomnography (PSG) measurements:
   1. Mean latency to persistent sleep ≥20 min (with neither of the two nights <15 min)
   2. Mean wake after sleep onset ≥30 min (with neither of the two nights <20 min)
   3. Mean TST <420 min
10. In women of childbearing potential: negative plasma pregnancy test at Visit 1; negative urine pregnancy test at Visit 3; consent to undertake pregnancy tests up to 30 days after completion of treatment; and agreement to use contraception with a prescribed method starting from the screening period until at least 30 days after the end of treatment.

*Exclusion Criteria*

Subjects who met any of the following criteria were excluded from the study:

1. Current sleep disorders other than insomnia (such as sleep apnea syndrome), or a history of related respiratory disorders, periodic limb movement disorder, restless legs syndrome, circadian rhythm sleep disorder, rapid eye movement sleep behavior disorder, or narcolepsy
2. Self-reported afternoon nap time of ≥1 hour per day, and on ≥3 days per week
3. Daily caffeine intake ≥600 mg
4. Subjects planning to work at night within the 2-week period before the screening visit or who plan to work at night during the study
5. Subjects who have traveled across three or more time zones within the week prior to the screening visit, or plan to travel across three or more time zones during the study
6. Pregnant, planning pregnancy, or lactating
7. Subjects who are deemed by the (sub)investigator to have clinically relevant deviations from normal values in hematological or biochemical tests
8. Aspartate aminotransferase and/or alanine aminotransferase >3 times above the upper limit of normal (ULN) and/or total bilirubin >2 times above the ULN (except those with a history of Gilbert’s syndrome)
9. Unstable medical conditions, serious medical disorders, or acute illnesses within 1 month before Visit 1 that are deemed by the (sub)investigator to affect the safety of the patient or evaluation of the clinical study
10. Systolic blood pressure >150 mmHg and/or diastolic blood pressure >90 mmHg
11. Resting pulse rate <50 bpm or ≥100 bpm
12. Any of the following conditions associated with the corrected QT (QTc) interval:
    1. QT interval prolongation (QTc >450 ms): if QTc >450 ms on the first electrocardiogram (ECG), ECG was repeated ≥30 min later on the same day. If on the second ECG QTc >450 ms, the subject was ineligible;
    2. History of risk factors for torsade de pointes (heart failure, hypokalemia, family history of long QT syndrome, etc.)
13. Any of the following conditions associated with suicidal tendencies:
    1. Intentional suicidal ideation during screening, with or without planning (persons who answered “yes” to question 4 or 5 on the Columbia-Suicide Severity Rating Scale)
    2. History of suicide attempts
14. Known factors or illnesses that may affect compliance with drug medication, study procedures, or interpretation of results, such as mental illness, a history of noncompliance with medication regimens, or not being willing to comply with clinical study requirements
15. Receiving other investigational drugs within 1 month prior to Visit 1
16. Hypersensitivity or contraindication to drugs in the same class as the study drug or excipients in the study drug formulation
17. Use of prohibited central nervous system drugs, including over-the-counter medications and Chinese herbal medicines, from five half-lives of the applicable drugs (and at least 2 weeks) before Visit 1 until 24 h after the final dose of the study drug
18. Subjects who plan to start new cognitive behavioral therapy within 1 month prior to Visit 3
19. Use of moderate or potent cytochrome P450 3A4 inhibitors or inducers from 1 week prior to Visit 1 until 24 h after the last dose of the study drug
20. Consumption of grapefruit and/or Seville orange (including squeezed juice) from 1 week before Visit 1 until 24 h after end of treatment
21. Diagnosis of alcohol or substance abuse or dependence within 2 years prior to the screening visit, or subjects who cannot refrain from alcohol intake for at least three consecutive days
22. Positive urine drug test results (benzodiazepines, barbiturates, cannabinoids, opiates, amphetamines, MDMA [ecstasy], or cocaine) or positive alcohol breath tests
23. Heavy smoking (≥10 cigarettes per day) and/or the inability to refrain from smoking for ≥14 h during the night
24. Apnea–hypopnea index ≥15 events per hour according to the American Academy of Sleep Medicine (AASM) criteria on the first night of PSG screening
25. Apnea or hypopnea events (according to AASM criteria) and pulse oximetry oxygen saturation <80% on the first night of PSG screening
26. Periodic limb movement in arousal index ≥15 per hour on the first night of PSG screening
27. Other subjects who were judged to be ineligible by the principal or clinical investigator

**Figure S1. Phase 1 study design**

**
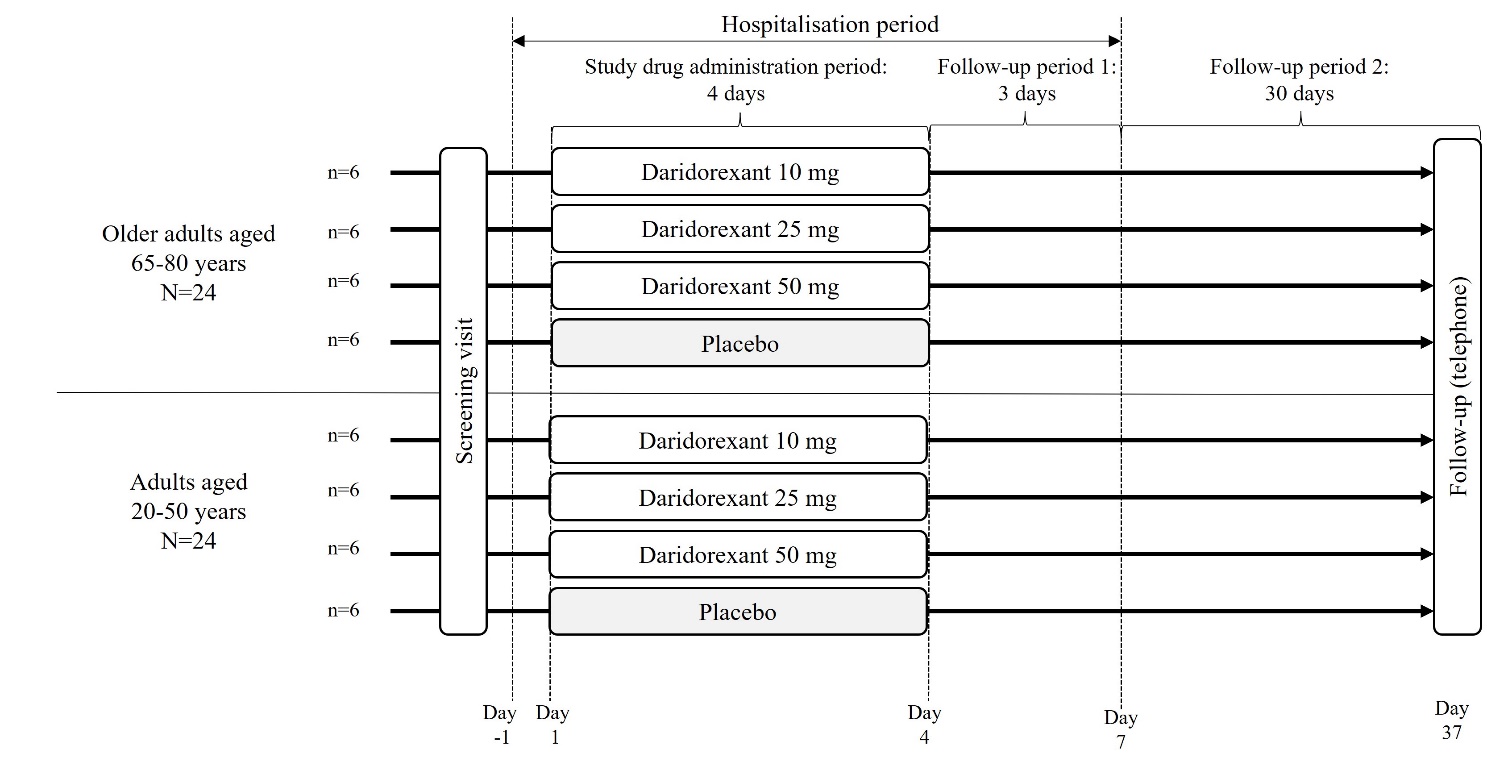
**

The screening period lasted from Day −24 to Day −2. Screening test and review of inclusion and exclusion criteria were performed during the period from 21 days before the start of administration of the study drug (Day −21) to 2 days before (Day −2) in men and women of non-childbearing potential and during the period from 24 days before the start of administration of the study drug (Day −24) to Day −5 in women of childbearing potential.

During each of the four treatment periods, pharmacokinetic blood samples were collected as follows:

Day 1 predose, and at 0.5 h, 1 h, 1.5 h, 2 h, 3 h, 4 h, 6 h, 8 h, 10 h, 12 h, and 24 h (=predose on Day 2).

Day 2 and Day 3 predose.

Day 4 predose, and at 0.5 h, 1 h, 1.5 h, 2 h, 3 h, 4 h, 6 h, 8 h, 10 h, 12 h, 24 h, 48 h, 72 h.

**Figure S2. Phase 2 study flow diagram**

**
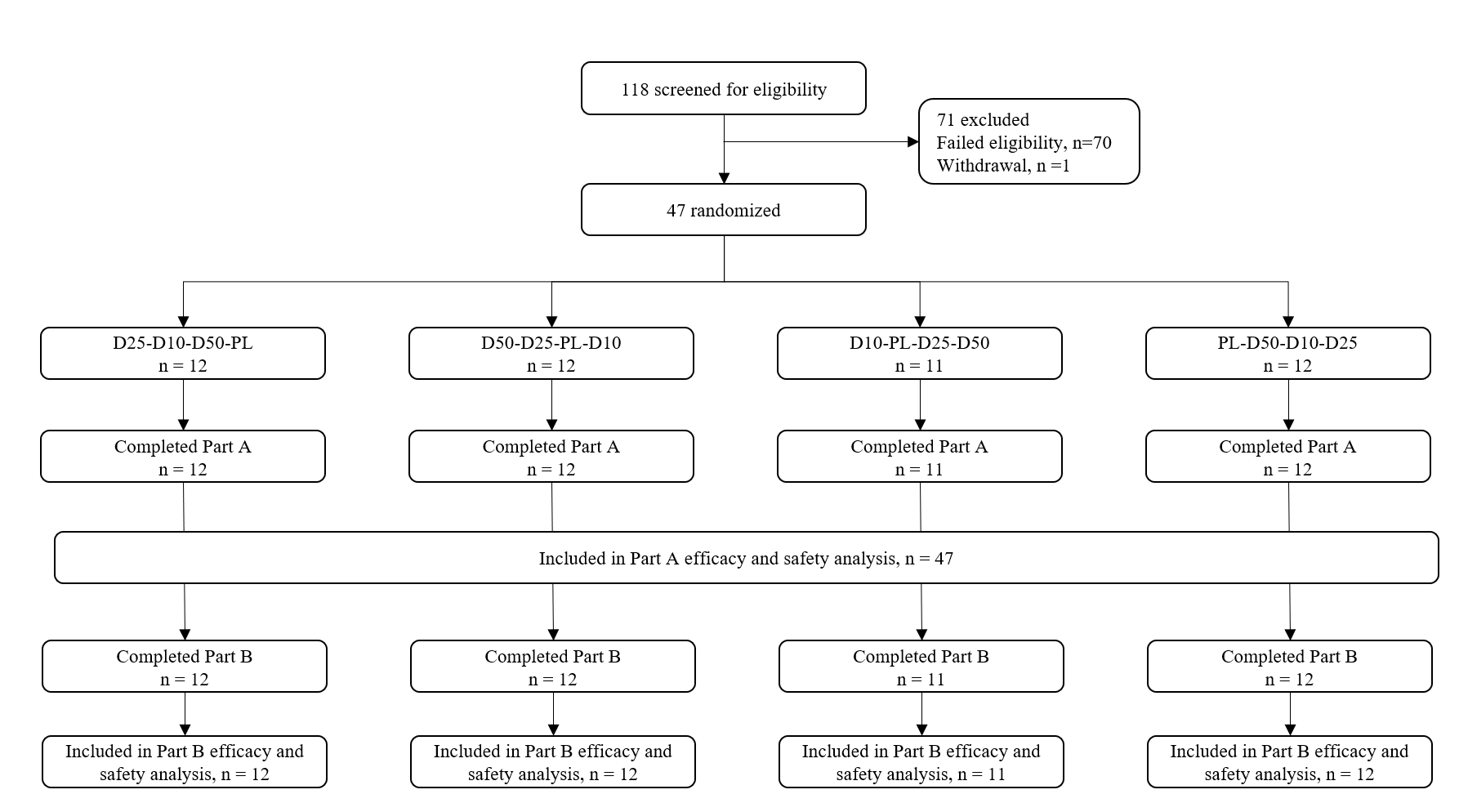
**

D10 = daridorexant 10 mg; D25 = daridorexant 25 mg; D50 = daridorexant 50mg; PL = placebo.

**Table S1. Baseline demographics of the Phase 1 study**

|  | **Daridorexant dose** | | |  |  |
| --- | --- | --- | --- | --- | --- |
| **Overall population** | **10 mg**  **(n = 12)** | **25 mg  (n = 12)** | **50 mg  (n = 12)** | **Placebo  (n = 12)** | **Overall  (N = 48)** |
| Sex, n (%)  Female  Male | 7 (58.3)  5 (41.7) | 7 (58.3)  5 (41.7) | 5 (41.7)  7 (58.3) | 5 (41.7)  7 (58.3) | 24 (50.0)  24 (50.0) |
| Mean age, years (range) | 48.3  (21, 78) | 47.6  (20, 72) | 49.9  (21, 74) | 47.4  (21, 72) | 48.3  (20, 78) |
| Mean body weight, kg (range) | 56.1  (43.5, 67.3) | 54.3  (42.8, 64.4) | 60.9  (48.2, 73.4) | 56.9  (47.0, 72.0) | 57.1  (42.8, 73.4) |
| Mean height, cm (range) | 161.5  (149.7, 174.3) | 159.2  (145.1, 175.1) | 163.8  (148.2, 177.7) | 162.4  (150.4, 173.2) | 161.7  (145.1, 177.7) |
| Mean body mass index, kg/m^2^ (range) | 21.5  (18.7, 24.7) | 21.4  (19.2, 24.9) | 22.6  (19.5, 24.5) | 21.6  (19.2, 24.9) | 21.8  (18.7, 24.9) |

**Table S2. Summary of main pharmacokinetic parameters (overall population) in healthy Japanese subjects (Phase 1 study)**

| **Overall population** | | **Daridorexant dose** | | |
| --- | --- | --- | --- | --- |
| **PK parameter** | **Timepoint** | **10 mg**  **(n = 12)** | **25 mg**  **(n = 12)** | **50 mg**  **(n = 12)** |
| t_max_ (h) | Day 1 | 1.00  [0.5, 3.0] | 0.50  [0.5, 3.0] | 0.75  [0.5, 3.0] |
|  | Day 4 | 0.50  [0.5, 1.5] | 0.50  [0.5, 2.0] | 1.00  [0.5, 3.0] |
| C_max_ (ng/mL) | Day 1 | 307.45  (241.34, 391.67) | 762.55  (661.85, 878.57) | 938.94  (715.10, 1232.86) |
|  | Day 4 | 408.37  (328.29, 507.98) | 746.96  (589.27, 946.84) | 1089.41  (882.88, 1344.24) |
| AUC_0-24_ (h·ng/mL) | Day 1 | 1585.72  (1382.11, 1819.33) | 3786.94  (3297.12, 4349.52) | 6453.23  (5253.30, 7927.24) |
|  | Day 4 | 1881.27  (1561.02, 2267.23) | 4279.47  (3701.68, 4947.46) | 6614.71  (5576.02, 7846.87) |
| t_1/2_ (h) | Day 1 | 7.70  (5.96, 9.93) | 8.23  (6.31, 10.73) | 8.05  (6.78, 9.56) |
|  | Day 4 | 7.88  (6.22, 9.98) | 7.65  (6.30, 9.30) | 7.42  (6.10, 9.04) |
| AI | Day 4/Day 1 | 1.20  [0.9, 1.5] | 1.11  [0.9, 1.3] | 1.04  [0.7, 1.3] |

Data are expressed as geometric mean (95% confidence interval) except for the accumulation index (arithmetic mean
[range]) and for t_max_ which is expressed as median [range]. N = 11 at 25 mg on Day 4.
AI, accumulation index (based on AUC_0-24_); AUC_0-24,_ area under the plasma concentration–time curve from time 0 to 24 h; C _max_, maximum plasma concentration; PK, pharmacokinetic; t_1/2_, terminal half-life_;_ t_max_, time to reach C_max_.

**Table S3. Summary of treatment-emergent AEs in healthy subjects (Phase 1 study)**

|  | **Daridorexant dose** | | |  |
| --- | --- | --- | --- | --- |
| **Overall population** | **10 mg (n = 12)** | **25 mg  (n = 12)** | **50 mg (n = 12)** | **Placebo  (n = 12)** |
| Subjects reporting ≥ 1 AE, n (%) | 6 (50.0) | 11 (91.7) | 11 (91.7) | 3 (25.0) |
| Somnolence | 6 (50.0) | 11 (91.7) | 11 (91.7) | 2 (16.7) |
| Vertigo positional | 0 | 1 (8.3) | 0 | 0 |
| Increased ALT | 0 | 0 | 0 | 1 (8.3) |
| Increased AST | 0 | 0 | 0 | 1 (8.3) |

AE, adverse event; ALT, alanine aminotransferase; AST, aspartate aminotransferase.

**Table S4. Change from baseline to Days 1/2 in WASO (min) by quarter of the night in subjects with insomnia disorder (Phase 2 study)**

|  |  |  | **Daridorexant dose** | | |
| --- | --- | --- | --- | --- | --- |
| **Mean WASO, min (SD)** | | **Placebo** | **10 mg** | **25 mg** | **50 mg** |
|  |  |  |  |  |  |
| Overall (n = 47) | Baseline | 84.2 (40.5) | 84.2 (40.5) | 84.2 (40.5) | 84.2 (40.5) |
|  | Days 1/2 | 61.2 (34.1) | 46.2 (23.7) | 41.2 (24.4) | 33.3 (29.7) |
|  | Change at Days 1/2 | -23.0 (38.0) | -38.0 (43.1) | -43.0 (34.6) | -50.9 (30.7) |
|  |  |  |  |  |  |
| Q1 (n = 43) | Baseline | 10.8 (7.6) | 10.8 (7.6) | 10.8 (7.6) | 10.8 (7.6) |
|  | Days 1/2 | 8.6 (7.8) | 5.3 (4.1) | 4.8 (3.9) | 3.2 (2.7) |
|  | Change to Days 1/2 | -2.0 (10.1) | -5.5 (8.2) | -6.2 (7.5) | -7.6 (8.3) |
|  |  |  |  |  |  |
| Q2 (n = 47) | Baseline | 17.8 (17.4) | 17.8 (17.4) | 17.8 (17.4) | 17.8 (17.4) |
|  | Days 1/2 | 10.5 (8.8) | 8.9 (7.4) | 6.8 (6.2) | 5.9 (5.0) |
|  | Change to Days 1/2 | -7.3 (18.9) | -8.9 (17.0) | -11.0 (19.0) | -11.9 (15.3) |
|  |  |  |  |  |  |
| Q3 (n = 47) | Baseline | 19.3 (16.3) | 19.3 (16.3) | 19.3 (16.3) | 19.3 (16.3) |
|  | Days 1/2 | 14.1 (15.6) | 9.7 (9.6) | 9.0 (8.7) | 7.1 (9.2) |
|  | Change to Days 1/2 | -5.2 (18.2) | -9.6 (18.3) | -10.3 (15.1) | -12.2 (14.4) |
|  |  |  |  |  |  |
| Q4 (n = 47) | Baseline | 37.7 (24.9) | 37.7 (24.9) | 37.7 (24.9) | 37.7 (24.9) |
|  | Days 1/2 | 28.2 (22.7) | 22.4 (18.2) | 20.6 (17.0) | 17.1 (18.8) |
|  | Change to Days 1/2 | -9.5 (24.5) | -15.4 (23.3) | -17.1 (21.7) | -20.6 (21.0) |

Changes from baseline to Days 1/2 by quarter of the night (Q1, 2, 3, 4) in wake after sleep onset (WASO; minutes) in patients with insomnia disorder receiving daridorexant 10 mg, 25 mg, 50 mg or placebo. Days 1 and 2 refers to the mean value of the corresponding 2 polysomnography treatment nights for a given treatment period.

SD, standard deviation; Q, quarter; WASO, wake after sleep onset.

**Table S5. Change from baseline to Days 1/2 in WASO by prespecified subgroup in subjects with insomnia disorder (Phase 2 study)**

| **Mean (SD)** |  |  | **Daridorexant dose** | | |
| --- | --- | --- | --- | --- | --- |
|  |  | **Placebo** | **10 mg** | **25 mg** | **50 mg** |
| Overall (n = 47) | Baseline | 84.2 (40.5) | 84.2 (40.5) | 84.2 (40.5) | 84.2 (40.5) |
|  | Days 1/2 | 61.2 (34.1) | 46.2 (23.7) | 41.2 (24.4) | 33.3 (29.7) |
|  | Change to Days 1/2 | -23.0 (38.0) | -38.0 (43.1) | -43.0 (34.6) | -50.9 (30.7) |
| Sex |  |  |  |  |  |
| Female (n = 25) | Baseline | 84.7 (50.6) | 84.7 (50.6) | 84.7 (50.6) | 84.7 (50.6) |
|  | Days 1/2 | 62.3 (34.9) | 42.8 (19.5) | 41.1 (27.8) | 36.0 (37.8) |
|  | Change to Days 1/2 | -22.4 (45.8) | -41.9 (51.3) | -43.6 (42.5) | -48.7 (33.0) |
|  |  |  |  |  |  |
| Male (n = 22) | Baseline | 83.7 (26.0) | 83.7 (26.0) | 83.7 (26.0) | 83.7 (26.0) |
|  | Days 1/2 | 60.0 (33.8) | 50.2 (27.6) | 41.4 (20.6) | 30.3 (16.8) |
|  | Change to Days 1/2 | -23.6 (27.7) | -33.5 (31.9) | -42.3 (23.7) | -53.4 (28.5) |
| Age |  |  |  |  |  |
| <52 years (n = 22) | Baseline | 81.6 (48.1) | 81.6 (48.1) | 81.6 (48.1) | 81.6 (48.1) |
|  | Days 1/2 | 60.2 (30.0) | 44.7 (20.3) | 45.3 (27.1) | 37.7 (38.7) |
|  | Change to Days 1/2 | -21.5 (43.4) | -36.9 (52.3) | -36.3 (41.4) | -43.9 (27.8) |
|  |  |  |  |  |  |
| ≥52 years (n = 25) | Baseline | 86.5 (33.4) | 86.5 (33.4) | 86.5 (33.4) | 86.5 (33.4) |
|  | Days 1/2 | 62.2 (37.9) | 47.6 (26.6) | 37.7 (21.8) | 29.5 (18.8) |
|  | Change to Days 1/2 | -24.3 (33.5) | -38.9 (34.1) | -48.8 (26.9) | -57.0 (32.4) |
| ISI category |  |  |  |  |  |
| 15-21 (n = 34) | Baseline | 85.0 (44.5) | 85.0 (44.5) | 85.0 (44.5) | 85.0 (44.5) |
|  | Days 1/2 | 57.2 (33.1) | 44.0 (18.6) | 37.7 (21.7) | 31.5 (31.0) |
|  | Change to Days 1/2 | -27.8 (37.2) | -41.0 (45.1) | -47.3 (33.2) | -53.4 (28.6) |
|  |  |  |  |  |  |
| ≥22 (n = 13) | Baseline | 82.2 (28.9) | 82.2 (28.9) | 82.2 (28.9) | 82.2 (28.9) |
|  | Days 1/2 | 71.8 (35.7) | 52.0 (33.9) | 50.3 (29.4) | 38.0 (26.8) |
|  | Change to Days 1/2 | -10.4 (38.8) | -30.1 (-37.9) | -31.8 (36.9) | -44.2 (36.0) |

Changes from baseline to Days 1/2 in wake after sleep onset (WASO) in patients with insomnia disorder receiving daridorexant 10 mg, 25 mg, 50 mg or placebo by prespecified subgroup. Days 1/2 refers to the mean value of the corresponding 2 polysomnography treatment nights for a given treatment period.

ISI, insomnia severity index; SD, Standard deviation; WASO, wake after sleep onset.

**Table S6. TST and percentage of TST spent in each sleep stage over the night (Phase 2 study)**

|  |  |  | **Daridorexant dose** | | |
| --- | --- | --- | --- | --- | --- |
| **Mean (SD)** |  | **Placebo  (n = 47)** | **10 mg**  **(n = 47)** | **25 mg  (n = 47)** | **50 mg  (n = 47)** |
| TST, overall, min | Baseline | 349.6 (53.9) | 349.6 (53.9) | 349.6 (53.9) | 349.6 (53.9) |
|  | Days 1/2 | 402.4 (39.8) | 420.3 (26.5) | 426.3 (26.9) | 437.3 (32.1) |
| % TST in each sleep stage: | |  |  |  |  |
| Stage N1 | Baseline | 20.1 (11.0) | 20.1 (11.0) | 20.1 (11.0) | 20.1 (11.0) |
|  | Days 1/2 | 17.1 (7.9) | 15.3 (7.5) | 14.9 (7.2) | 13.7 (7.3) |
|  |  |  |  |  |  |
| Stage N2 | Baseline | 49.1 (9.1) | 49.1 (9.1) | 49.1 (9.1) | 49.1 (9.1) |
|  | Days 1/2 | 50.0 (6.8) | 49.3 (7.4) | 49.0 (6.8) | 47.7 (6.5) |
|  |  |  |  |  |  |
| Stage SWS | Baseline | 11.2 (8.1) | 11.2 (8.1) | 11.2 (8.1) | 11.2 (8.1) |
|  | Days 1/2 | 11.1 (8.0) | 11.3 (7.7) | 11.0 (7.8) | 11.5 (7.3) |
|  |  |  |  |  |  |
| Stage REM | Baseline | 19.6 (6.3) | 19.6 (6.3) | 19.6 (6.3) | 19.6 (6.3) |
|  | Days 1/2 | 21.8 (5.9) | 24.0 (6.0) | 25.0 (5.8) | 27.1 (5.5) |

Days 1/2 refers to the mean value of the corresponding 2 polysomnography treatment nights for a given treatment period.

REM, rapid eye movement; SD, standard deviation; SWS, slow wave sleep; TST, total sleep time.

**Table S7. Sleep efficiency measured by PSG (Phase 2 study)**

|  |  |  | **Daridorexant dose** | | |
| --- | --- | --- | --- | --- | --- |
| **Mean (SD)** |  | **Placebo  (n=47)** | **10 mg (n=47)** | **25 mg   (n=47)** | **50 mg  (n=47)** |
| Sleep efficiency, % | Baseline | 72.8 (11.2) | 72.8 (11.2) | 72.8 (11.2) | 72.8 (11.2) |
|  | Days 1 and 2 | 83.8 (8.3) | 87.6 (5.5) | 88.8 (5.6) | 91.1 (6.7) |

Days 1/2 refers to the mean value of the corresponding 2 polysomnography treatment nights for a given treatment period.

PSG, polysomnography; SD, standard deviation

**Table S8. Total DSST, KSS-J and SDS scores (Phase 2 study)**

|  |  |  | **Daridorexant dose** | | |
| --- | --- | --- | --- | --- | --- |
| **Mean (SD)** |  | **Placebo** | **10 mg** | **25 mg** | **50 mg** |
| **Part A** | N | 47 | 47 | 47 | 47 |
| Total DSST score | Baseline | 75.1 (10.1) | 75.1 (10.1) | 75.1 (10.1) | 75.1 (10.1) |
|  | Days 1/2 | 85.9 (10.8) | 87.1 (11.8) | 86.0 (12.3) | 86.2 (11.3) |
|  |  |  |  |  |  |
| KSS-J Score | Baseline | 5.2 (1.9) | 5.2 (1.9) | 5.2 (1.9) | 5.2 (1.9) |
|  | Days 1/2 | 4.3 (1.7) | 4.5 (1.6) | 4.5 (1.6) | 4.7 (1.7) |
|  |  |  |  |  |  |
| SDS score | Baseline | 10.8 (7.9) | 10.8 (7.9) | 10.8 (7.9) | 10.8 (7.9) |
|  | Days 1/2 | 9.0 (7.9) | 9.1 (7.4) | 8.2 (7.4) | 8.8 (7.4) |
| **Part B** | N | 12 | 12 | 12 | 11 |
| Total DSST score | Baseline | 72.1 (10.0) | 75.0 (8.9) | 76.8 (11.1) | 76.6 (11.1) |
|  | EOT | 89.5 (12.7) | 91.8 (7.5) | 100.5 (13.6) | 90.7 (14.5) |
|  |  |  |  |  |  |
| KSS-J Score | Baseline | 5.2 (2.0) | 4.7 (2.1) | 5.7 (2.0) | 5.3 (1.4) |
|  | EOT | 3.7 (1.9) | 3.0 (1.9) | 3.1 (1.7) | 4.3 (2.2) |
|  |  |  |  |  |  |
| SDS score | Baseline | 9.8 (8.0) | 8.9 (8.2) | 12.4 (7.5) | 12.4 (8.3) |
|  | EOT | 5.2 (5.6) | 4.9 (6.0) | 7.9 (6.0) | 9.6 (7.0) |

Data presented as mean (SD). Absolute values at baseline, Days 1/2 and EOT in DSST, KSS-J, and SDS scores in patients with insomnia disorder receiving daridorexant 10 mg, 25 mg, 50 mg or placebo.

Baseline is the mean value of the two overnight PSG measurements during the run-in period; Days 1/2 are the mean values of two overnight PSG measurements; EOT is the measurement in the morning of EOT.

DSST, Digit Symbol Substitution Test; EOT, end of treatment; KSS, Karolinska Sleepiness Scale; PSG, polysomnography; SD, standard deviation; SDS, Sheehan Disability Scale.
